# Effects of highly selective sympathectomy on neurogenic bowel dysfunction in spinal cord injury rats

**DOI:** 10.1038/s41598-021-95158-5

**Published:** 2021-08-05

**Authors:** Peipei Xu, Shuang Guo, Yang Xie, Zitong Liu, Changbin Liu, Xin Zhang, Degang Yang, Huiming Gong, Yixin Chen, Liangjie Du, Yan Yu, Mingliang Yang

**Affiliations:** 1 School of Rehabilitation Medicine, Capital Medical University, No. 10, Jiao Men Bei Lu, Fengtai District, Beijing, 100068 China; 2 China Rehabilitation Research Center, Beijing, 100068 China; 3 Center of Neural Injury and Repair, Beijing Institute for Brain Disorders, Beijing, 100068 China; 4 China Rehabilitation Science Institute, Beijing, 100068 China; 5 Rehabilitation Department, Ruijing Hospital, Shanghai Jiaotong University School of Medicine, No. 197 Ruijin Er Lu, Huangpu District, Shanghai, 200001 China; 6 Department of Rehabilitation Medicine, Beijing Tiantan Hospital, Capital Medical University, No. 119 Sihuan West Road, Fengtai District, Beijing, 100070 China

**Keywords:** Spinal cord diseases, Spinal cord diseases

## Abstract

Neurogenic bowel dysfunction, including hyperreflexic and areflexic bowel, is a common complication in patients with spinal cord injury (SCI). We hypothesized that removing part of the colonic sympathetic innervation can alleviate the hyperreflexic bowel, and investigated the effect of sympathectomy on the hyperreflexic bowel of SCI rats. The peri-arterial sympathectomy of the inferior mesenteric artery (PSIMA) was performed in T8 SCI rats. The defecation habits of rats, the water content of fresh faeces, the intestinal transmission function, the defecation pressure of the distal colon, and the down-regulation of Alpha-2 adrenergic receptors in colon secondary to PSIMA were evaluated. The incidence of typical hyperreflexic bowel was 95% in SCI rats. Compared to SCI control rats, PSIMA increased the faecal water content of SCI rats by 5–13% (P < 0.05), the emptying rate of the faeces in colon within 24 h by 14–40% (P < 0.05), and the defecation pressure of colon by 10–11 mmHg (P < 0.05). These effects lasted for at least 12 weeks after PSIMA. Immunofluorescence label showed the secondary down-regulation of Alpha-2 adrenergic receptors after PSIMA occurred mainly in rats’ distal colon. PSIMA mainly removes the sympathetic innervation of the distal colon, and can relieve the hyperreflexic bowel in rats with SCI. The possible mechanism is to reduce the inhibitory effect of sympathetic activity, and enhance the regulatory effect of parasympathetic activity on the colon. This procedure could potentially be used for hyperreflexic bowel in patients with SCI.

## Introduction

Neurogenic bowel dysfunction (NBD), the loss of normal bowel function caused by nerve problems, is a particularly common occurrence in patients with spinal cord injury (SCI), spina bifida, and multiple sclerosis. In the clinic, two types of NBD, the hyperreflexic bowel and the areflexic bowel, which secondary to upper motor neuron injury, and conus medullaris and cauda equina injury, respectively, are observed^1^. Nearly half of SCI patients are troubled by NBD. NBD's impact on the daily life of patients with SCI is second only to walking impairment and urinary dysfunction^2^. At present, there are a variety of methods for clinical treatment of NBD, including optimized diet management, manual stimulation of defecation, transanal irrigation^3^, and drug treatment, such as laxatives, oral gastrointestinal motility drugs, rectal suppositories, etc.^4,5^. For severe NBD, surgical procedures, such as sacral nerve root stimulation^6–9^ and colostomy^10^ have been attempted. Here, we hope to develop a new surgical method that can relieve the severe NBD caused by SCI as a supplement.

Sympathetic and parasympathetic divisions typically function in opposition to each other. Excessive activation of sympathetic nerve and/or suppression of parasympathetic nerve in colon generally results in typical NBD, which characteristic with colonic motility dysfunction (usually impairs propagation of luminal contents, and is consequently associated with slow-transit constipation)^11^, excessive absorption of water in the faeces by the colon, and hardened stool, etc. Sympathectomy has been used to treat pain, sweat gland secretion disorders, vascular diseases and so on^12^. Previous studies in normal rats demonstrated that colonic transmission movement could be accelerated by cutting off the external sympathetic nerves, and slowed down by excising parasympathetic nerves^13,14^. We hypothesize that for patients with the hyperreflexic bowel secondary to cervical and thoracic SCI, partial removal of the sympathetic innervation of the left colon may decrease the sympathetic effects, relatively enhance the regulation of parasympathetic nerve to the colon, and help to alleviate the NBD after SCI.

**Figure 1:**
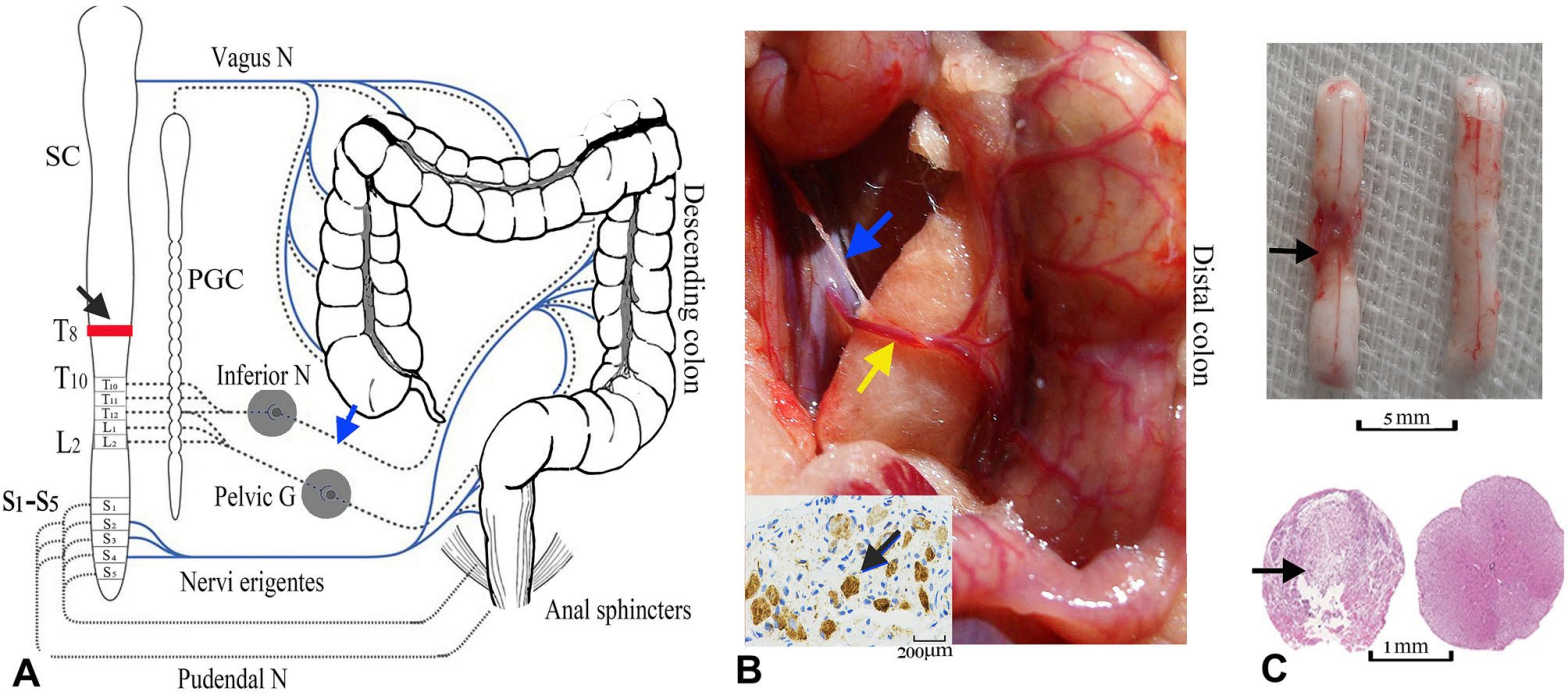
Surgery of SCI model and PSIMA. (**A**) Design of PSIMA for humans (the anatomy of colon), the sympathetic innervation (T10-L2) and parasympathetic innervation (S2–S4) are showed by dashed lines and blue lines, respectively. The blue arrow indicates the site of the nerve cut, and the black arrow indicates the level of the SCI. (**B**) The anatomy of accompanying sympathetic plexus and inferior mesenteric artery in a rat. The inferior mesenteric artery (indicated by yellow arrow), which originates from the anterior wall of the bifurcation of the left and right common iliac arteries, is mainly divided into the upper, middle, and lower branches, and with its accompanying sympathetic plexus (indicated by blue arrow) is situated to the distal colon. Lower left panel, an excised sympathetic nerve specimen stained with TH staining. (**C**) The upper panel showed the damaged segment of the spinal cord, four weeks after SCI (left) with the normal control (right), the lower panel, HE staining under the optical microscope, showed a complete damage at a cross section of the damaged segment. SC (spinal cord), PGC (paravertebral ganglia chain), Inferior N (inferior mesenteric ganglia), Pelvic G (pelvic ganglion), Vagus G (vagus ganglion), Pudendal G (pudendal ganglion), T8 and T10 (the 8th and 10th thoracic levels of the spinal cord), L2 (the second lumbar level of the spinal cord).

The sympathetic innervation of the left colon, mainly from the accompanying mesenteric plexus of the inferior mesenteric artery^15,16^, is anatomically very easy to identify and surgically remove. Therefore, the peri-arterial sympathectomy of the inferior mesenteric artery (PSIMA) (Fig. 1A), which only cuts off sympathetic postganglionic neurons, has potential clinical value and feasibility in the treatment of NBD in patients with SCI.

The sympathetic innervation of the rat’s distal colon is similar to that of human left colon (Fig. 1B)^17^. This study intends to observe the effect of the PSIMA on the NBD of T8 SCI rats (at the eighth thoracic segment).

## Results

### NBD in SCI rats

In this study, an NBD model secondary to a T8 complete SCI was established. The SCI rats with typical NBD were given PSIMA (Fig. 1). The mortality rate of rats within four weeks of surgery was 8%. It is noteworthy that 20% of the surgical rats were excluded from the study, mostly because of death by anaesthesia, abdominal and back wound infection, pressure ulcers, and urinary tract infection.

Three days after the SCI surgery, or PSIMA treatment, each group has 12 qualified rats for the study, whose Basso, Beattie and Bresnahan (BBB) locomotor scale scores of the SCI were less 1 point and had no wound infection. Two weeks later, the SCI and the SCI with S group, both, has 10 qualified rats.

The Basso, Beattie and Bresnahan (BBB) locomotor scale scores of the SCI group were 0.10 ± 0.21, 9.70 ± 0.35, and 10.90 ± 0.83 points at three days, 4 weeks, and 12 weeks after surgery, respectively (normal rats scored 20–21 points)^18^. The experimental group (SCI rats with S) were 0.10 ± 0.31, 9.65 ± 0.34, and 11.10. ± 0.93 points, respectively. No significant difference between the groups was observed. In the NBD model, a bulging abdomen, anal spasm, dry and hard stools, were often observed. The incidence of typical NBD was 95% in the SCI model.

### PSIMA alleviates constipation

We evaluated the effect of PSIMA on the normal rats and SCI rats from the aspects of defecation rate, the number of faecal particles excreted during an observation period, and Bristol Stool Scale. In normal rats, the effect is manifested by increased bowel frequency and mild diarrhoea. This effect is obvious within 3–5 days after PSIMA, and returns to its normal state in about two weeks. In SCI rats with PSIMA, beneficial effects were observed, the defecation rate reduced from 72 to 57% (p < 0.05, Chi-square test), the stool of some rats changed from Bristol grade 1–2 (dry and hard) to grade 3–4 (normal) (p < 0.05, Chi-square test), the number of faecal particles excreted in a given 2-h period increased from 2.5 ± 1.2 to 4.2 ± 1.9 (p < 0.05, two-way ANOVA). These effects lasted for at least 12 weeks after PSIMA in SCI rats, which the defecation rate was about 56% and the faecal particles excreted 4.3 ± 1.9 (Table 1).

**Table 1:** Ratings of constipation.

Groups	Total	Sampling	Evaluation time (after surgery or enrol)	Defecation rate		Number of faecal particles		Bristol stool scale		
				2-h (%)	24-h (%)	2-h	24-h	1–2	3–4	5–7
Intact rats	n = 18	n = 12	3 days	42	100	4.8 ± 2.0	48.9 ± 10.9	0	13	2
			2 weeks	44	100	4.6 ± 2.2	49.9 ± 11.6	2	12	2
			4 weeks	39	100	4.5 ± 2.3	40.2 ± 8.9	0	13	1
			12 weeks	44	100	4.7 ± 3.0	47.7 ± 10.1	1	14	1
				**42**	**100**	**4.7 ± 2.8**	**46.7 ± 9.1**			
Rats with S	n = 18	n = 12	3 days	33	100	4.2 ± 2.1	44.9 ± 11.9	0	6	6
			2 weeks	42	100	4.7 ± 2.4	49.9 ± 9.9	0	15	0
			4 weeks	42	100	4.3 ± 2.7	47.3 ± 12.9	0	14	1
			12 weeks	44	100	5.0 ± 3.1	48.9 ± 8.9	1	13	2
				**40.2**^‡^§	**100**	**4.5 ± 2.9**^‡^	**47.7 ± 10.9**	^‡^§	^‡^§	
SCI rats	n = 18	n = 10	3 days	64	100	2.6 ± 1.7	43.6 ± 11.5	10	10	3
			2 weeks	81	100	2.8 ± 1.3	45.7 ± 13.5	19	8	2
			4 weeks	69	100	2.3 ± 1.1	41.3 ± 9.5	15	8	2
			12 weeks	75	100	2.4 ± 1.2	40.2 ± 12.5	17	9	1
				**72**^†^	**100**	**2.5 ± 1.2**^†^	**42.7 ± 12.1**	^†^	^†^	
SCI rats with S	n = 18	n = 10	3 days	50	100	4.5 ± 2.1	43.6 ± 11.5	4	12	2
			2 weeks	61	100	4.2 ± 2.1	42.6 ± 12.8	6	14	2
			4 weeks	61	100	3.9 ± 1.8	41.9 ± 9.5	6	12	4
			12 weeks	56	100	4.3 ± 1.9	40.6 ± 11.0	8	12	0
				57**	**100**	4.2 ± 1.9*	**42.1 ± 11.3**	**	**	

Defecation rate, the percentage of defecation rats during a given period. Bold type, the average of the four time points. *p < 0.05, comparison between SCI Rats with S and SCI Rats; **p < 0.05, between SCI Rats with S and Intact rats; ^†^p < 0.05, between SCI Rats and Intact Rats; ^‡^p < 0.05, between Rats with S and SCI Rats; ^§^P < 0.05, between Rats with S and SCI Rats with S (Chi-square test or two-way ANOVA).

### PSIMA promotes the response of SCI rats to manual defecation stimulation

We designed a manual method for stimulating bowel movements in rats, and observed the incidence of defecation, the defecation reaction time, and the number of faecal particles excreted etc. induced by the manual methods. PSIMA could reduce the incidence of defecation (67.5% vs 47.5%, p < 0.05, Chi-square test), shorten the defecation reaction time (2.3 ± 1.2 vs 1.6 ± 1.2 s), and increase the number of faecal particles excreted in a given period (1.2 ± 1.2 vs 2.4 ± 1.2, p < 0.05, two-way ANOVA) in SCI rats (Table 2).

**Table 2:** Response of defecation induced by manual defecation stimulation.

Groups	Sampling	Evaluation time (weeks)	Defecation rate (%)	Defecation reaction time (min)	Number of faecal particles
Intact rats	n = 12	4	40	1.6 ± 1.2	2.3 ± 0.6
		12	42	1.4 ± 1.5	2.3 ± 1.2
			**41**	**1.5 ± 1.3**	**2.35 ± 1.1**
Rats with S	n = 12	4	42	1.5 ± 1.4	2.5 ± 1.3
		12	42	1.3 ± 1.0	2.4 ± 1.4
			**42**^‡^	**1.4 ± 1.3**	**2.45 ± 1.2**^‡^
SCI rats	n = 10	4	70	2.3 ± 1.1	1.1 ± 1.2
		12	65	2.2 ± 1.5	1.3 ± 1.2
			**67.5**^†^	**2.3 ± 1.2**	**1.2 ± 1.2**^†^
SCI rats with S	n = 10	4	50	1.5 ± 0.9	2.3 ± 1.1
		12	45	1.7 ± 1.2	2.4 ± 1.5
			47.5*	**1.6 ± 1.2**	2.4 ± 1.2*

Defecation reaction time, the time from the beginning of the massage stimulation to that the first faecal particle was excreted. Bold type, the average of the two time points. *p < 0.05, comparison between SCI Rats with S and SCI Rats; ^†^p < 0.05, between SCI Rats and Intact Rats; ^‡^p < 0.05, between Rats with S and SCI Rats (Chi-square test or two-way ANOVA).

### PSIMA increases faecal moisture

**Figure 2:**
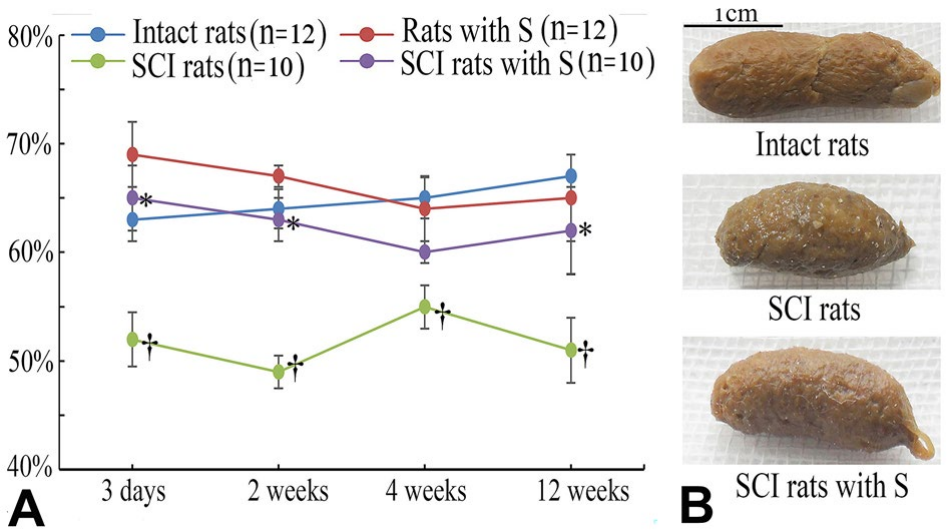
Faecal water content. In (**A**), *SCI rats with S vs SCI rats, ^†^SCI Rats versus Intact Rats, P < 0⋅05 (two-way ANOVA). (**B**) Normal rats exclude fresh faecal particles that are soft, moisturized, and cracked. SCI rats exclude fresh faeces that are hard and firm and have a tight surface texture.

In rats, constipation occurred 3–5 days after SCI. Fresh stools in SCI rats were mostly dry and hard granules with a water content of approximately 50–55% (in normal rats, 63–67%). The stools became softer and the water content increased, especially 3–5 days after PSIMA. PSIMA increased the water content of SCI rat’s faeces to 60–65% (p < 0.05, two-way ANOVA, Fig. 2A). This effect could last for at least 12 weeks after PSIMA.

### PSIMA enhances colon transmission function

**Figure 3:**
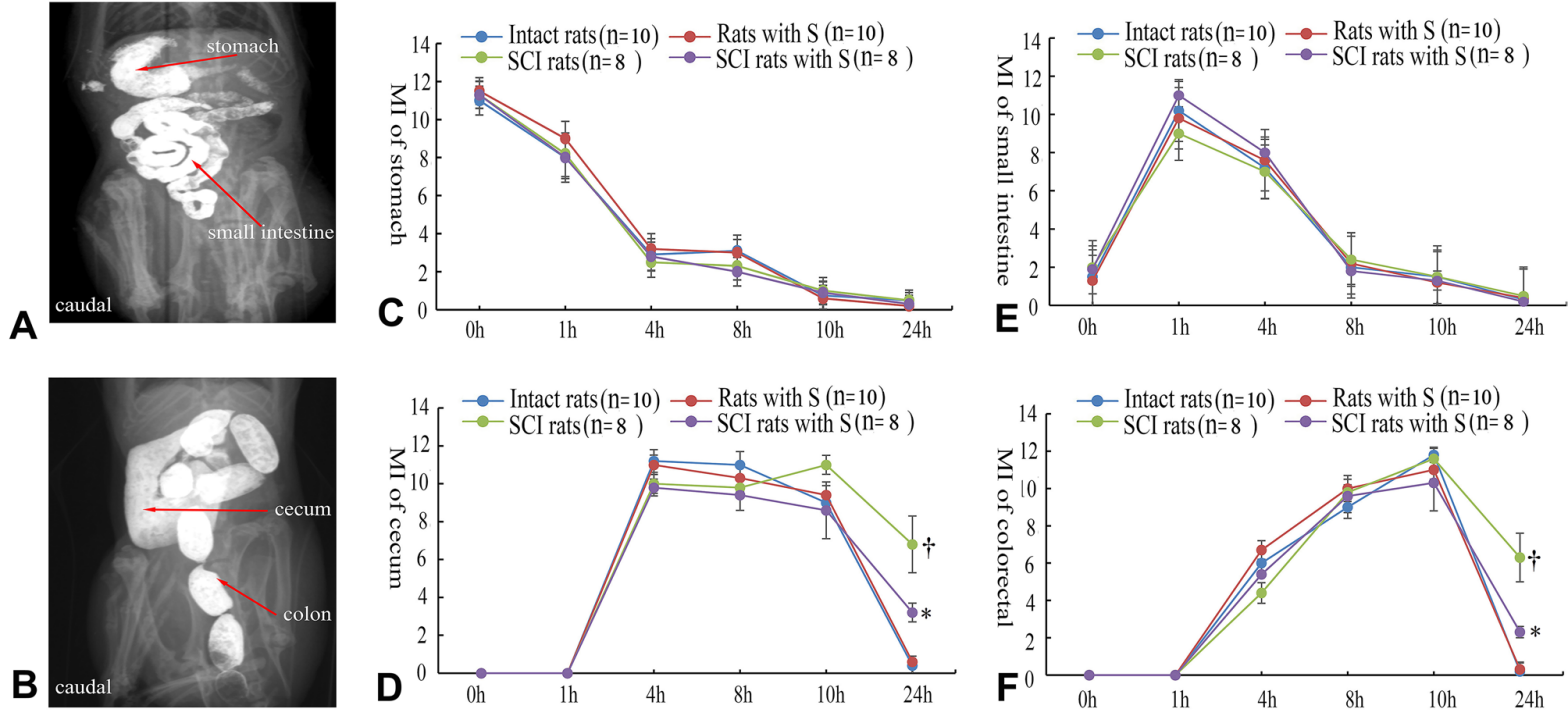
Barium imaging and Motility index (MI) of digestive tract. (**A**) and (**B**) Division of digestive tract, from an intact rat. (**C**–**F**) MI of digestive tract at four weeks after surgery, *SCI rats with S vs SCI rats, ^†^SCI Rats vs Intact Rats, P < 0⋅05 (two-way ANOVA). In the semi-quantitative analysis, the total score was 12, higher scores and rapidly changes indicate more motility. MI showed the differences in the colon between SCI rats with S and SCI rats.

The small intestine appeared within 1 h, and the cecum appeared within 4 h. Within 8 h, granular faeces appeared in the colon. The number of granular faeces in the colon reached a peak within 8–10 h. At the set observation time, there were no significant differences in the imaging rates of the stomach and small intestine between the observation groups. Imaging rate roughly reflects the speed of intestinal transmission of intestinal contents from the proximal end to the distal end. At two, four, and 12 weeks after surgery, the emptying rate of faecal particles in the colon of rats within 24 h: normal rats were 60% (6/10), 67% (6/9), and 75% (6/8); Rats with S were 70% (7/10), 67% (6/9), 75% (6/8); SCI rats were 40% (4/10), 56% (5/9), and 50% (4/8); SCI rats with PSIMA were 80% (8/10), 70% (7/10), 75% (6/8). PSIMA increases the rate of faecal emptying in the colon of SCI rats within 24 h (total rate, Rats with S (13/27) vs SCI rats with PSIMA (21/28), p < 0.05, Chi-square test). We performed a semi-quantitative analysis of the stomach, small intestine, cecum, and colorectal area^19,20^ (Fig. 3A, B). The transmission function impairment caused by T8 SCI mainly occurred in the colon. The evaluation results showed similar conclusions (Fig. 3C–F).

### PSIMA promotes bowel movement in SCI rats

**Figure 4:**
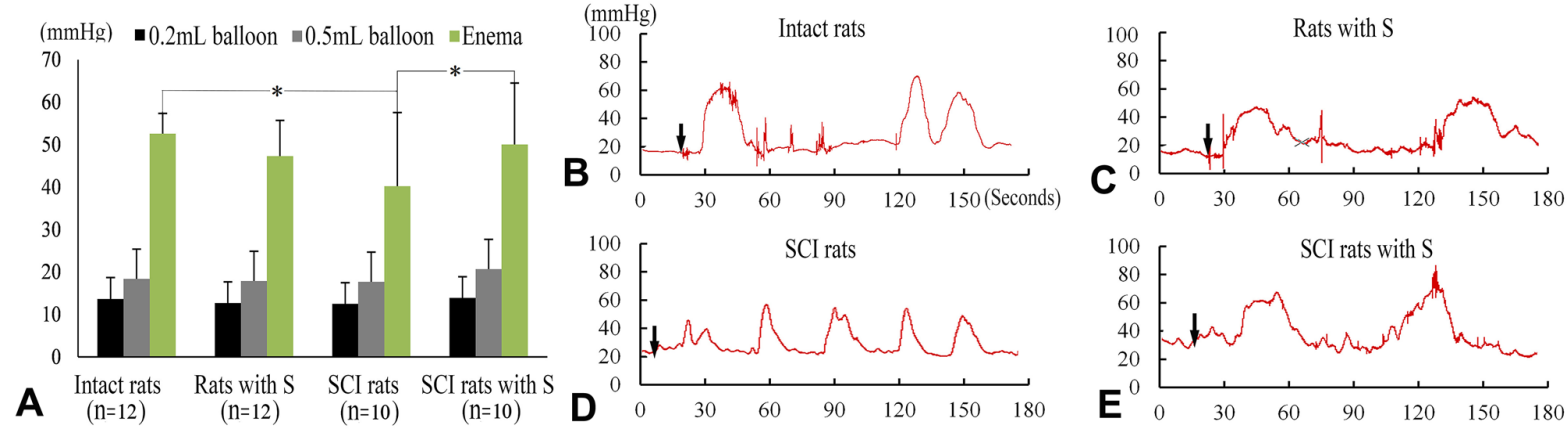
Distal colon pressure at four weeks after surgery. (**A**) Comparison of pressures among three pressure measurement modes. *Intact rats vs SCI rats and SCI rats with S versus SCI rats, P < 0⋅05 (two-way ANOVA). (**B**–**E**) Defecation reflexes, while the defecation reflexes occurred, the pressure waves were irregular (**B**, **C**, and **E**), but a rhythmic oscillation could occur in SCI rats (**D**). Black arrows indicate the time taken to give glycerin enema.

In the balloon non-expansion mode, the average value of each group was 12–14 mmHg, which roughly reflected the internal pressure of the distal colon without the faecal filling in the colon, and there were no significant differences between the groups (Fig. 4A). In the balloon expansion mode (filled to a volume of 0.5 mL), the average value of each group was 17–20 mmHg, which roughly reflected the internal pressure of the distal colon under the retention of granular faeces in the colon. There was no significant difference between the groups (Fig. 4A). During the pressure measurement, low-intensity irregular colon contraction waves could be observed in 50% of normal rats (6/12), in 55% of normal rats with S (6/11), in 60% of SCI rats with S (6/10), and in 20% of SCI rats (2/10).

Stronger defecation reflexes, usually, presented with high-intensity irregular colon contraction waves, were activated by glycerin enemas in each group (Fig. 4B–E). The corresponding pressure waves, with 25–60 mmHg wave amplitude, 10–40 s duration, appeared within 10–30 s after defecation administration. The maximum defecation pressure was 30–80 mmHg, which varies greatly between individuals. Normal rats had the highest defecation pressure and often push the pressure balloon out of the anus. Compared with the SCI group, the defecation pressure of SCI rats with S increased by an average of about 10 mmHg (40.1 ± 11.0 vs 50.3 ± 10.9, P < 0.05, two-way ANOVA) at four weeks after the surgery, close to the normal group level 52.3 ± 4.5 mmHg. At 12 weeks after surgery, the increase of the defecation pressure was about 11 mmHg (42.2 ± 14.5 vs 53.2 ± 16.4 mmHg, n = 6).

### PSIMA mainly down-regulates Alpha-2 adrenergic receptors in distal colon

**Figure 5:**
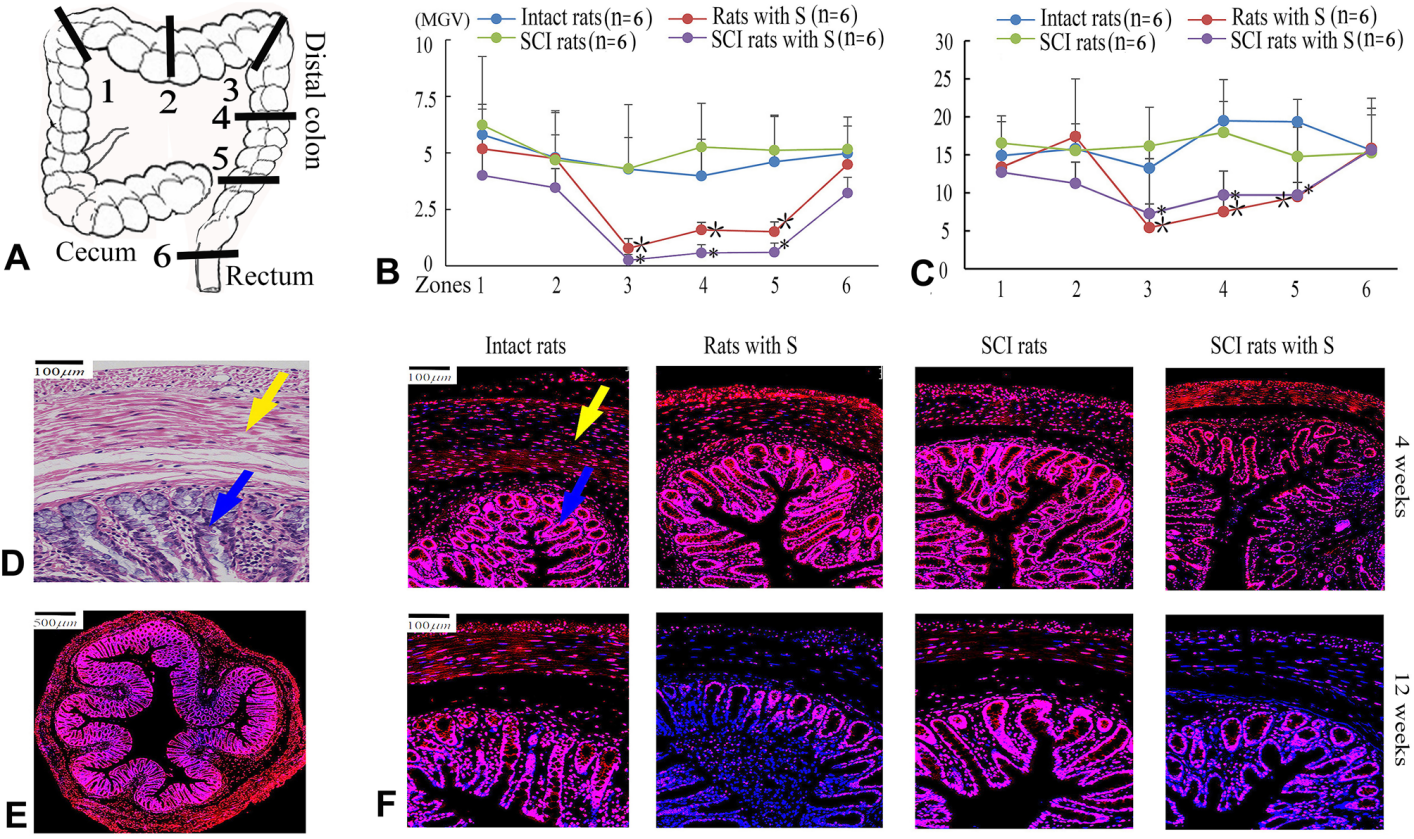
Distribution of Alpha-2 adrenergic receptor in colon. (**A**) The excised sections were showed by zones. (**B**, **C**) The mean grayscale value (MGV) of Alpha-2 adrenergic receptors in colonic smooth muscle layer (**B**) and colonic mucosa layer (**C**) 12 weeks after PSIMA. *SCI rats with S versus SCI rats, ^†^Rats with S versus Intact rats in Zone 3, 4, 5, P < 0⋅05 (two-way ANOVA). (**D**) Colonic tissue (TRI staining), the smooth muscle tissues are directed by the yellow arrows, and the colonic mucosa tissue by the blue arrows. (**E**, **F**) Representative fluorescent images of Alpha-2 adrenergic receptors (red) in colonic tissue, cross-sectional anatomy of colon (**E**), and high-power images (**F**).

Colonic smooth muscle cells, mucosal columnar epithelial cells, and glandular epithelial cells all distribute Alpha-2 adrenergic receptors, and are particularly abundant in colonic glandular epithelial cells. The mean grayscale values (MGV) of receptor expression in the mucosa layer of colon zone 3, 4, 5 at 12 weeks were 13.3–19.4 MGV in intact rats, 14.8–17.9 MGV in SCI rats, 5.4–8.4 MGV in rats with S, and 7.3–9.7 MGV in SCI rats with S. The significant change of Alpha-2 adrenergic receptors in quantity occurred in zone 3, 4 and 5 in the rats with the PSIMA (P < 0.05, two-way ANOVA) (Fig. 5).

## Discussion

The anatomy of the rats’ colon, which has a highly developed cecum, is slightly different from that in human beings^21^, and is divided into a proximal and distal colon in most literatures. The description, the proximal and distal colon, is suitable for a rat in prone position, but the arrangement of rat’s colon becomes similar to that in human beings while a rat is in a supine position. There is species differences in the innervation of colon, for example, the vagal innervation runs the entire length of the colon in rodents, but it ends in colonic splenic flexure in human beings^22,23^. The sympathetic innervation in colon in rodents is not clearly understood^24^. However, the sympathetic nerves usually accompany with arteries into their targeting tissues^25^. It could be speculated from the blood vessel distribution that the denervation caused by PSIMA should mainly occur in the distal colon in rats and in the left colon in human beings. After the PSIMA, the secondary down-regulation of Alpha-2 adrenergic receptors, mainly within the distal colon, confirmed our guess. PSIMA does not reduce the parasympathetic activity in rat’s colon. The expected effect of PSIMA is to reduce the sympathetic activity, relatively strengthen the parasympathetic activity, and thereby promote the motility and secretion of the rat colon to alleviate NBD.

Our results showed that the water content of fresh faeces, the amount of faeces excreted in a given period, and the colonic faecal emptying rate within 24 h increased after PSIMA; indicating that PSIMA can improve the symptoms of constipation in SCI rats. Near 5–10% change of water content of faecal particles was found in SCI rats after PSIMA, which could significantly affect the physical properties of faecal particles, such as hardness and shape. Aside from these expected effects of PSIMA, the secondary effects resulting from the changes in the physical properties of the faeces might play an important role in relieving constipation. More mucus, the faeces appeared shinier and moister (Fig. 2B), was secreted after PSIMA. The secreted mucus adhered to the surface of the granular faeces to reduce the water loss of the faeces and the frictional resistance between the faeces and the intestinal wall. Our results also showed that mild diarrhoea could appear in normal rats after PSIMA, and returns to the pre-operative normal states in two weeks, indicating a spontaneous intestinal regulation ability in rats. We thought that similar spontaneous intestinal regulation processes should exist in SCI rats and after PSIMA, which may play beneficial compensatory roles or may lead to new disorders. These problems require more in-depth research.

We observed that in SCI rats with PSIMA, the number of faecal particles excreted increased and the defecation rate reduced in a given 2-h period. The increase in the number of faecal particles excreted implies an improvement in NBD of SCI rats. Normal rats defecate at any time and the defecation frequency increase after SCI. The increase may be related to the inability to discharge stool, which stays in the colon to frequently stimulate bowel movements. Whether the decrease of defecation rate in SCI rats with PSIMA implies an improvement or a deterioration of symptoms requires further study.

We found that the activation of effective defecation reflex needed a stronger trigger (glycerin enema) and its intensity also decreased in SCI rats. These changes can neither be attributed to the disability of the intestinal smooth muscle itself, nor to the disruption of the defecation reflex arc in our NBD MODEL. In fact, the intestinal smooth muscle is often compensatory hypertrophy, which is thought to be secondary to myogenic hyperactivity after SCI, and the defecation reflex arc is basically intact in its signal path in the model of T8 SCI rats. The suppression of effective defecation reflex implied there existed sympathetic hyperactivity in the model of the hyperreflexic bowel.

Two modes, non-expansion mode (0.1 mL) and expansion mode (0.5 mL) were used to investigate the tension response of the distal colon to mechanical distension. The non-expansion mode roughly reflected the internal pressure of the distal colon without the faecal filling (or empty distal colon). The expansion mode roughly reflected the internal pressure of the distal colon under the retention of granular faeces. No differences between the complete transected rats and uninjured control rats were found. The possible reason was the invalidity of the model / pressure choice. However, no difference in the tension response to electrical stimulation in distal colon were also reported in a recent study, which used the same T8 transected SCI rats^26^. These findings imply that the distal colons in T8 SCI rats could maintain a similar basic distension response just like that in the intact rats.

The tension response also showed that an involuntary rhythmic contraction of the colon (Fig. 4D), which is inefficient to defecation, usually occurred in SCI rats, could be activated by the mild stimulation (0.5 mL balloon). However, under strong stimulation of the defecation agent, glycerin enema, the defecation reflex in SCI rats is close to that of normal rats. This result is consistent with clinical observations. Patients with SCI have difficulty with bowel movements, but the administration of defecation agents can cause rapid and effective bowel movements. Therefore, it may be one of the most important neurological mechanisms for NBD in SCI that the sympathetic and parasympathetic nerves cannot collaborate with one another in the colon, usually, with sympathetic hyperactivity and/or parasympathetic hypoactivity.

In clinic, patients with SCI can benefit from an appropriate tone of the pelvic floor musculature and the external sphincter, which might prevent the fecal incontinence, leaking unexpectedly from the rectum. In our study, we assumed that the sympathetic innervation of colon is similar between rats and human beings. Based on the range of secondary down-regulation of Alpha-2 adrenergic receptors, we confirmed that the PSIMA mainly involved in the sympathetic innervation of the rat’s distal colon. Hence, the aspect of external anal sphincter activity is not likely to be affected by the experimental therapy.

The potential impacts of PSIMA on gastrointestinal reflex and neurogenic bladder requires further study in the model of SCI rats. In previous study, the bilateral hypogastric nerve transection could alleviate the neurogenic bladder dysfunction in SCI rats. From the perspective of autonomic nerve regulation, it should have a promoting effect on gastrointestinal reflex, and be beneficial for the neurogenic bladder, particularly with urethral sphincter spasm, which we aim to study in future.

## Conclusion

In summary, The PSIMA cutting off sympathetic postganglionic neurons is highly selective, different from the most sympathetic surgeries, which are usually involved in the sympathetic chain, sympathetic node. PSIMA can improve NBD, partially in SCI rats. The possible mechanism is by reducing sympathetic activity, relatively increase the regulation of parasympathetic activity on the colon. Its potential surgical indications are suitable for NBD with sympathetic hyperactivity and/or parasympathetic hypoactivity. As it is very easy and minimally invasive to perform laparoscopically, PSIMA has potential clinical value in the treatment of NBD in patients with SCI.

## Materials and methods

Sprague–Dawley rats (n = 72, 6–7 weeks old, weighing 180–250 g, including males and females) were used in this study, with the approval of the Experimental Animal Centre of Capital Medical University of China (Approval No. AEEI-2019-045), and was conducted according to the ethical rules of the Animal Experiments and Experimental Animal Welfare Committee. All procedures in this study were conducted in compliance with the ARRIVE guidelines^27^.

### NBD model and surgery

Rats were given general anaesthesia (5% isoflurane anaesthetics for rapid induction, 2% isoflurane maintenance), and placed on a heating pad with far-infrared warming in prone position. The T_7–9_ laminae were removed to expose the spinal cord, and the spinal cord was completely transacted using surgical scissors at the T_8_ level^28^. The neurological injury was evaluated using the BBB locomotor score^18^. Three days post-surgery, the bowel function of SCI rats was evaluated. The SCI rats whose faeces were Faecal Bristol grade 1–2^29^, and/or abdomens were bulging with clogged stools in the colon were diagnosed as typical NBD.

PSIMA was conducted in normal rats and SCI rats with typical NBD three days after the SCI. Rats were in supine for PSIMA. An incision, about 2–3 cm long, was performed along the midline of the abdomen. After opening the abdominal cavity, the distal colon was laterally retracted, revealing the inferior mesenteric artery as well as its accompanying sympathetic plexus (Fig. 1B). The accompanying sympathetic plexus was carefully dissected and excised without damaging the blood vessels using an operating microscope.

The study design divided rats into four groups, namely; the normal control group (Intact rats, n = 18), the control group only subjected to PSIMA (Rats with S, n = 18, “with S” means with surgery of the PSIMA), the SCI group (SCI rats, n = 18), and the experimental group subjected to SCI and PSIMA (SCI Rats with S, n = 18).

All the rats were housed in individual cages under controlled temperature and light conditions (temperature 21 ± 1℃, humidity 30–70%, with a 12-h light–dark cycle), they had access to food and water ad libitum, and were fed a uniformly processed feed (China feed formula, LAD 1000), for the entire duration of the experiment. There wasn't an administration of pain medication after surgery. The manual bladder compression was usually given for 1–2 weeks to manage secondary urinary retention in SCI rats after surgery.

### Evaluation of constipation

Defecation habits and faecal properties were evaluated three days, two weeks, four weeks, and 12 weeks after surgery (SCI or PSIMA). The faecal particles excreted by rats during a two hour and a 24-h period were investigated, and Bristol Stool Scale^29^ was blindly performed by two people according to the physical properties of the faeces (shape, colour, level of dryness, hardness, etc.), which are a qualitative description in Bristol Stool Scale. These data were collected in the morning (before the manual bladder compression) once a day for three consecutive days.

Defecation reflex was induced by massage stimulation on abdomen and evaluated four and 12 weeks after surgery. Rat abdomens were massaged, clockwise for 30 s with the thumb. The rat’s defecation reflex was then observed and the number of faecal particles excreted during a five minute period was recorded, once a day for three consecutive days.

### Measurement of faecal water content

Fresh faecal water content was determined at three days, two weeks, four weeks, and 12 weeks after surgery. Before collecting the faeces, rats were prohibited from eating and drinking for two hours. Each time the rat expels faeces, it is immediately collected and sealed in a plastic bag. The total amount of faeces excreted by the rat within a total of two hours, were weighed, then dried in a constant temperature drying oven (90 °C) for 48 h, and weighed again. The formula for calculating the water content is; water content (%) = [(wet weight-dry weight)/wet weight] × 100%.

### Evaluation of intestinal transmission function

The evaluation time points were at two, four, and 12 weeks after surgery. After fasting for 2 h, the rats were orally administered 2.5 ml of BaSO4 (2 g/ml, temperature 22 °C) without anaesthesia. Abdominal X-rays were taken at 0 (within 5 min), 1, 2, 4, 8, 10, and 24 h after the oral administration to observe the presence of contrast agent in the digestive tract. At the time of filming, the rats were placed in special cages, which restrict their activity, and abdominal X-rays were taken (voltage 60 kV, current 10 mA, shooting focal length 100 cm, exposure time 0.06 s). The digestive tract is divided into four areas: stomach, small intestine, cecum, and colorectal (Fig. 3A, B). The image data was analysed by a trained imaging expert using an image analysis system, ImageJ 1.38 for Windows (National Institute of Health, USA, free software: http://rsb.info.nih.gov/ij/), for semi-quantitative scoring to obtain the motility index (MI) of digestive tract, as shown in previous studies^19,20^. The focus of this assessment is the delivery of contrast media in the colon.

### Determination of distal colon pressure

Distal colon pressure was measured by the pressure balloon. The evaluation time points were at four- and twelve-week post-surgery. Rats were first treated with inhalation general anaesthesia in a special cage. The two-way Foley catheter (Size, 8F, 2.7 mm in diameter, with one balloon port and one drainage port) was used to measure the distal colon pressure. The balloon of the catheter was placed in the distal colon (3–5 cm deep from anus), and a 3-way medical valve was used to connect the balloon port and the pressure monitor (Powerlab 8/20 data acquisition system, AD Instruments Pty Ltd). After the rats woke up, two modes were used to measure the pressure; (1) Non-expansion mode: Inject 0.1 ml saline through the three-way valve to fill the balloon to obtain pressure; (2) Expansion mode: Inject 0.5 ml of saline rapidly through the three-way valve, to inflate the balloon and stimulate the distal colon (the balloon was 0.5 mL in volume and 5–8 mm in diameter, which is similar to the size of granular faeces in the colon). Finally, 0.1 mL Glycerin Enema (0.625 g glycerol per mL) was quickly injected into the distal colon through the drainage port of the catheter to activate the defecation reflex.

### Histopathological observation

The surgically removed sympathetic nerve tissue was stained with anti-tyrosine hydroxylase (TH) to confirm whether the excised tissue was the target tissue.

The injured segment of the spinal cord was excised for hematoxylin–eosin staining (HE) to observe the degree of SCI. The colon segment, about 1 cm at length, was excised 3–5 cm away from the anus, and then stained with histochemical staining for collagen and smooth muscle (Masson’s Trichrome stain, TRI).

### Immunofluorescence labelling of Alpha-2 adrenergic receptor

The sympathetic nerve regulates intestinal smooth muscle and mucosal glands mainly through Alpha-2 adrenergic receptors^30^. We observed the expression of Alpha-2 adrenergic receptors in different parts of the colon. The rats were sacrificed to harvest the colon at 4 weeks and 12 weeks after the PSIMA. Six colon segments, each about 1 cm long (Fig. 5A), were cut. Continuous transverse sections were prepared. The immunofluorescence staining was performed for Alpha-2 adrenergic receptors. The primary antibody (rabbit anti-rat antibody, 1:200; Invitrogen, PAI-048) was added, and samples were incubated at 4 °C overnight^30^. These specimens were then incubated in the appropriate secondary antibody (Cy3-conjugated goat anti-rabbit antibody, 1:300; Servicebio, GB21303) at room temperature for 50 min. DAPI solution (Servicebio, PA-048) developed colour for 10 min. The fluorescent images were then scanned and analysed using Case Viewer 2.1 (3DHISTECH Ltd).

### Statistical analysis

Data are expressed as mean ± SD. Data were analysed using one-or two-way ANOVA with Tukey’s multiple comparisons test, two-tailed unpaired t test, and chi-square test. P < 0.050 was considered statistically significant. SPSS22.0 was used for statistical evaluation. Since this is a study with several endpoints, the p-values provided have to be interpreted carefully.

## Data Availability

The data that support the findings of this study are available from NCBI, but restrictions apply to the availability of these data, which were used under license for the current study, and so are not publicly available. However, data are available from the authors upon reasonable request and with permission of NCBI.
